# Analysis of dynamic texture and spatial spectral descriptors of dynamic contrast-enhanced brain magnetic resonance images for studying small vessel disease

**DOI:** 10.1016/j.mri.2019.11.001

**Published:** 2020-02

**Authors:** Jose Bernal, Maria del C. Valdés-Hernández, Javier Escudero, Linda Viksne, Anna K. Heye, Paul A. Armitage, Stephen Makin, Rhian M. Touyz, Joanna M. Wardlaw

**Affiliations:** a Centre for Clinical Brain Sciences, University of Edinburgh, Edinburgh, UK; b School of Engineering, University of Edinburgh, Edinburgh, UK; c Academic Unit of Radiology, University of Sheffield, Sheffield, UK; d Institute of Cardiovascular and Medical Sciences, University of Glasgow, Glasgow, UK

**Keywords:** Dynamic descriptors, Principal component analysis, Dynamic brain magnetic resonance image, Cerebral small vessel disease

## Abstract

Cerebral small vessel disease (SVD) comprises various pathological processes affecting small brain vessels and damaging white and grey matter. In this paper, we propose a framework comprising region of interest sampling, dynamic spectral and texture description, functional principal component analysis, and statistical analysis to study exogenous contrast agent distribution over time in various brain regions in patients with recent mild stroke and SVD features.We compared our results against current semi-quantitative surrogates of dysfunction such as signal enhancement area and slope. Biological sex, stroke lesion type and overall burden of white matter hyperintensities (WMH) were significant predictors of intensity, spectral, and texture features extracted from the ventricular region (*p*-value < 0.05), explaining between a fifth and a fourth of the data variance (0.20 ≤Adj.*R*^2^ ≤ 0.25). We observed that spectral feature reflected more the dysfunction compared to other descriptors since the overall WMH burden explained consistently the power spectra variability in blood vessels, cerebrospinal fluid, deep grey matter and white matter. Our preliminary results show the potential of the framework for the analysis of dynamic contrast-enhanced brain magnetic resonance imaging acquisitions in SVD since significant variation in our metrics was related to the burden of SVD features. Therefore, our proposal may increase sensitivity to detect subtle features of small vessel dysfunction. A public version of the code will be released on our research website.

## 1 Introduction

Cerebral small vessel disease (SVD) encapsulates multiple pathological processes disrupting the optimum functioning of perforating cerebral arterioles, capillaries, and some venules, resulting in grey matter (GM) and white matter (WM) damage [[1], [2], [3]]. SVD is a serious problem causing between 20% to 25% of strokes, up to 45% of dementias, and substantial cognitive, psychiatric, and physical disabilities. At a global scale, SVD may be leading to between three and four million new cases of stroke [4] and 16 million new cases of dementia per year^1^ [3]. Despite being a worldwide matter and governmental priority, little is known about its cause(s) since much of SVD is clinically silent and late [1,4]. Therefore, efforts for understanding the pathophysiological mechanisms surrounding SVD and developing techniques to characterise this disease are of global impact.

Dynamic contrast-enhanced magnetic resonance imaging (DCE-MRI) is commonly used to investigate endothelial dysfunction, a pathophysiological component thought to be associated with the SVD pathogenesis [6], as it permits detecting leakage in tissue and cerebrospinal fluid (CSF) spaces thought to be caused by an impaired blood-brain barrier (BBB) or blood-CSF barrier [[7], [8], [9]]. In this imaging modality, a series of acquisitions are taken before and after injecting a Gadolinium-based contrast agent intravenously to image its distribution through brain tissues over time. The contrast agent causes the relaxation time of water molecules to decrease in T1w. Therefore, its accumulation in the extracellular extravascular space with increased BBB impairment leads to increased signal enhancement.

In a recent work [10], changes in local signal variations in tissue and CSF cavities were quantitatively measured in pre- and post-contrast study and showed to vary with increased overall SVD and white matter hyperintensity (WMH) burden. In this work, we aim to study signal intensity fluctuation on the entire DCE-MRI sequence using established computer vision descriptors to determine whether specific variation patterns relate to the health of the patient and their suitability for acting as a surrogate measure of small vessel impairment.

We consider texture and spectral descriptors. Textures, which encode local signal changes within a region of interest, have been successfully used to characterise WMH in T2 FLAIR scans [11]; study pre- vs post-contrast differences in small vessel disease [10]; and classify breast lesions into benign and malignant, predict chemotherapy response, and diagnose prostate cancer [12] in DCE-MRI. Power spectra, the strength of frequency components into the overall signal, have been successfully applied in dynamic susceptibility contrast MRI to characterise neurophysiological and hemodynamic patterns of Alzheimer's disease [13], detect and characterise oscillations in blood oxygen level-dependent imaging reflecting network connectivity [14], and discern between conduct disorder and healthy subjects from resting functional MRI acquisitions [15]. We hypothesise that the application of these computer vision descriptors to DCE-MRI scans can identify tissue differences in brain regions that relate to the burden of SVD features.

We propose a framework to study contrast signal-time trajectory in healthy and pathological brain regions in DCE-MRI acquisitions. The proposal comprises region of interest sampling, dynamic texture and spatial spectral description, functional principal component analysis, and statistical group comparison and linear regression. The contributions of this work are: (i) we introduce a fully functional framework to analyse DCE-MRI acquisitions based on dynamic spectral and texture descriptors and (ii) we show an application of our framework to the study of DCE-MRI signals of a cohort (*n* = 42) with a wide range of SVD burden.

## 2 Materials and methods

**Fig. 1:**
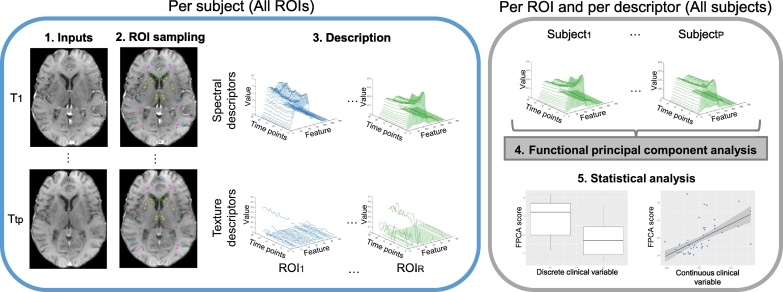
Scheme of our processing pipeline. The inputs are the DCE-MRI sequences (1). Initially, we process each case by sampling all regions of interest using an anatomically-relevant template (2), describing local signal variations in regions of interest using descriptors (3). Subsequently, we study the dynamic descriptors using functional principal component analysis (4) and statistical analysis (5). ROI: region of interest. FPCA: functional principal component analysis. *T*_1_,…,*T*_*TP*_: each time point of the DCE-MRIs. *Subject*_1_,…,*Subject*_*P*_: each patient in the cohort. *ROI*_1_,…,*ROI*_*R*_: each region of interest.

The pipeline consists of four steps, as shown in Fig. 1. First, we segmented all regions of interest for each patient in the cohort. Second, we described signals using dynamic spectral and texture descriptors. Third, we examined the resulting descriptions using a functional principal component analysis (FPCA). Fourth, we studied whether scores of the primary mode of variation were associated with any of the clinical variables. Details of each step are provided in the following sections.

### 2.1 Subjects and clinical variables

We used data from a prospective study of patients with recent mild stroke and SVD features (*n* = 42 subjects, 12 women, 19 lacunar stroke). Of 201 in the original study, we selected 42 on the basis of considering only high-quality scans (qualitative assessment of truncation and motion artefacts) and representing a wide spectrum of SVD feature burden, stroke lesion size, and index stroke lesion type (i.e. cortical vs lacunar). The sample clinical characteristics have been published previously [16,17]; those relevant to this work are condensed in Table 1. The baseline hypertension (y/n) defined as a previous history of hypertension, or hypertension diagnosed at presented of stroke, age, and percentages of WMH in intracranial volume were retrieved from the study database. Additionally, we considered a visual clinical rating recorded at inclusion, total SVD [18] score, to account for four neuroimaging features of the SVD (lacunes, microbleeds, perivascular spaces, and WMH).

**Table 1:** Distribution of demographics, risk factors, and imaging variables in our sample. Values in the right column correspond to the number of patients (percentage of the total). White matter hyperintensity values were normalised by the intracranial volumes.

Clinical variable	No. patients (% of the total)
Age	
[39, 49]	2 (5%)
(49, 59]	11 (26%)
(59, 69]	15 (36%)
(69, 79]	11 (26%)
(79, 89]	3 (7%)
Biological sex	
Male	30 (71%)
Female	12 (29%)
Hypertension	
Hypertensive	33 (79%)
Normotensive	9 (21%)
White matter hyperintensity volume	
[0.07%, 1.80%]	23 (55%)
(1.80%, 3.53%]	6 (14%)
(3.53%, 5.26%]	7 (17%)
(5.26%, 6.99%]	2 (5%)
(6.99%, 8.73%]	4 (9%)
Total small vessel disease score	
0	8 (19%)
1	10 (24%)
2	12 (28%)
3	7 (17%)
4	5 (12%)

Imaging was carried out on a 1.5 T MRI scanner (Signa HDxt, General Electric, Milwaukee, WI) using an eight-channel phased-array head coil. Both diagnostic and dynamic MR imaging acquisition parameters have been detailed in [19]. Diagnostic MRI at stroke presentation consisted of axial T2-weighted imaging (TR/TE = 6000/90 s, 24 × 24 cm field of view, 384 × 384 Propeller acquisition, 1.5 averages, 28 × 5 mm slices, 1 mm slice gap), axial fluid-attenuated inversion recovery imaging (TR/TE/TI = 9000/153/2200 ms, 24 × 24 cm field of view, 384 × 224 acquisition matrix, 28 × 5 mm slices, 1 mm slice gap), gradient echo imaging (TR/TE = 800/15 ms, 20° flip angle, 24 × 18 cm field of view, 384 × 168 acquisition matrix, 2 averages, 28 × 5 mm slices, 1 mm slice gap) and sagittal 3D T1-weighted imaging (inversion recovery-prepared spoiled gradient echo TR/TE/TI = 7.3/2.9/500 ms, 8° flip angle, 330 × 214.5 cm field of view, 256 × 146 acquisition matrix, 100 × 1.8 mm slices). DCE-MRI acquisitions were obtained at approximately one month after first stroke presentation and consisted of a 3D T1w spoiled gradient echo sequence with TR = 8.24 ms, TE = 3.1 ms, 24 × 24 cm FOV, reconstruction matrix 256 × 192 and 42 × 4 mm slices. Following a pre-contrast acquisition, an intravenous bolus injection of 0.1 mmol/kg of gadoterate meglumine (Gd-DOTA, Dotarem, Guerbet, France) was administered with the start of 20 further acquisitions with 12° flip angle and a temporal resolution of 73 s, leading to a DCE-MRI duration of about 24 min (≈21 time points).

### 2.2 Image processing and region-of-interest sampling

We performed all image analysis blindly to clinical and permeability data. We aligned all time points of the DCE-MRI acquisition to the 12° pre-contrast image to correct for bulk patient movement using FSL-FLIRT [20,21]. For determining the WMH volume percentage in intracranial volume, we applied a segmentation method that has been evaluated previously against manual annotations in images acquired with similar scanning protocols [16]. On 150 subjects, the average difference on ICV was 2.7% (95*%*CI = 7%). On 20 individuals, the Jaccard similarity coefficient for WMH was 0.61 (95*%*CI = ±0.37). In a test-retest analysis on 14 cases comprising volunteers and patients with mild non-disabling stroke, the coefficient of variation for repeated measurements of the segmentation technique was 0.21 [22]. Furthermore, trained analysts double-checked and manually edited these segmentation masks under the supervision of an experienced neuroradiologist.

**Fig. 2:**
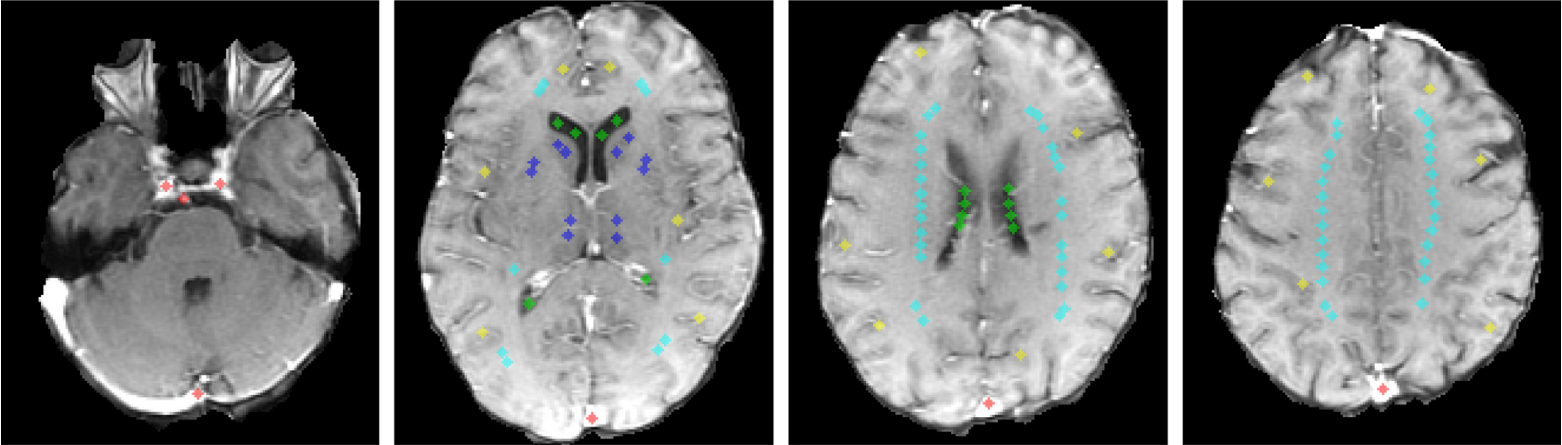
Example of selected regions of interest on four slices. From left to right, low, low-middle, middle-high, and high slices. Colour code is red, green, light blue, dark blue, and yellow for arteries and sagittal sinus, CSF, WM, GMD and GMC, respectively. (For interpretation of the references to colour in this figure legend, the reader is referred to the web version of this article.)

We sampled five brain regions, comprising blood vessels [BL], CSF, deep GM [GMD], cortical GM [GMC], and WM, using circular non-overlapping samples that covered approximately 12 mm^2^ in-plane and were distributed throughout four slices [[23], [24], [25]], as described in Table 2 and exemplified in Fig. 2. The figure illustrates four slices and sampling points for one of the patients in our cohort, but it does not indicate sampling points are fixed nor predetermined for every single patient. In fact, these spots vary to avoid areas with partial volume effects, evident contrast-enhanced related truncation artefacts, WMH, enlarged perivascular spaces, mineral depositions, lacunes and ischaemic or haemorrhagic lesions to avoid biasing our analysis. The samples were initially placed by a trained analyst using Analyze 11.0 (AnalyzeDirect Inc., Mayo Clinic), edited by another one, and, finally, agreed between both observers. We opted for sampling brain regions to reduce the influence of the spatial densities, partial volume effects, and avoid obvious correlations/associations as a result of descriptors encoding volumetric information [26].

**Table 2:** Slice and sampling point selection criteria. The number in parenthesis corresponds to the total number of sampling points per slice for a certain region of interest.

Slice	Criteria	Sampling points
Low	Must include brainstem, carotid arteries, basilar artery and superior sagittal sinus	Carotid arteries (1), basilar artery (1), and sagittal sinus (1)
Low-middle	Must include basal ganglia (i.e. caudate heads and lentiform nuclei), thalami, third ventricle, and horns of the lateral ventricles	Superior sagittal sinus (1), CSF (12), GMD (12), GMC (12), and WM (10)
Middle-high	Must be two-four slices above the previous slice in which basal ganglia are not visible, but centrum semiovale and ventricles	Superior sagittal sinus (1), CSF (8), GMC (6), and WM (28)
High	Must be the first or second slice above the ventricles	Superior sagittal sinus (1), GMC (6), and WM (28)

### 2.3 Description of regions of interest

#### 2.3.1 Power spectrum

We used the radial power spectrum (RPS) to account for spatial variations in the strength of the signal frequency components. First, we sampled the signal using the anatomical-relevant template. Second, we used the 3D discrete Fourier transform to obtain a representation of each volume in the frequency domain. Let *I* ∈ℝ^*N*×*N*×*N*^ be a brain MR volume, the corresponding discrete Fourier transform,

(1) $$F(u,v,w)=\underset{i,j,k}{\sum }I(i,j,k)\;\exp\left(-2\iota \pi \frac{ui+vj+wk}{N}\right).$$

Third, we computed the magnitude spectra and averaged all the frequencies over concentric rings of width 1 using the following formula

(2) $$R(r)=\frac{1}{{\left(2\pi \right)}^{2}}\int_{0}^{2\pi }\int_{0}^{2\pi }|F(r\;\sin(\theta )\cos(\phi ),r\;\sin(\theta )\sin(\phi ),r\cos(\theta ))|\;d\theta d\phi ,$$

where $r=\sqrt{u^{2}+v^{2}+w^{2}}$, $\theta =\cos^{-1}(w/r)$, and $\phi =\tan^{-1}(v/u)$ represent the corresponding spherical coordinates. For each time point and region of interest, signals were described using 256 frequencies.

#### 2.3.2 Grey-level co-occurrence matrix based descriptors

We measured local signal variations using metrics of homogeneity and variability extracted from grey-level co-occurrence matrices (GLCM). These matrices summarise the co-appearance of intensity values in an image, i.e. they quantify the frequency at which two intensity values occur in the same neighbourhood. Mathematically speaking, a co-occurrence matrix is calculated as follows:

(3) $$C(a,b)=\underset{x\in I}{\sum }\underset{y\in N(x)}{\sum }\left\{\begin{array}{c} 1\text{, if }x=a\wedge y=b\\ 0\text{, otherwise.} \end{array}\right)$$

where N(x) denotes the set of voxels in the neighbourhood of *x*. The matrix *C* reveals information of the region of interest. For instance, the higher the values in the diagonal, the more homogeneous the region under examination.

Haralick et al. [27] proposed various measures of homogeneity and heterogeneity of region of interest based on the normalised GLCM values. The process for computing them was four-fold. First, we quantised each region of interest in 2^4^ grey levels. Second, we computed the GLCM using an eight-connected neighbouring structure. Third, we normalised the GLCM by dividing each cell by the total number of voxel pairs. Fourth, we computed energy, contrast, correlation, variance, inverse difference moment, sum average, sum variance, sum entropy, and entropy [27], as condensed in Table 3. For each time point and each region of interest, the number of GLCM features per patient was 9.

**Table 3:** Considered texture descriptors based on the grey-level co-occurrence matrix. *C*_*a*+*b*_(*k*) represents the grey level sum distribution, expressed as *C*_*a*+*b*_(*k*) = ∑ _*a*_ ∑ _*b*_{*C*(*a*,*b*) if *a* + *b* = *k*,0 otherwise}. SA in the sum variance formula denotes the sum average operation.

GLCM metric	Brief description	Formula
Contrast	Overall contrast. The higher the co-occurrence of low and high intensity values, the higher the contrast.	∑ _*a*,*b*_|*a* − *b*|^2^*C*(*a*,*b*)
Correlation	Linear dependency between neighbouring voxels. Values close to 1 reflect high correlation.	$\sum_{a,b}\frac{\left(a-\mu_{a})(b-\mu_{b}\right)}{\sigma_{a}\sigma_{b}}C(a,b)$
Energy	Second angular moment measuring uniformity. Energy increases with increased homogeneity.	∑ _*a*,*b*_*C*^2^(*a*,*b*)
Variance	Dispersion of values around the mean. Variance increases with increased heterogeneity.	∑ _*a*_(*a* − *μ*_*a*_)^2^ ∑ _*b*_*C*(*a*,*b*)
Entropy	Measurement of randomness. Higher entropy values indicate higher heterogeneity.	$-\sum_{a,b}C(a,b)\log C(a,b)$
Inverse difference moment	Overall homogeneity. Higher values indicate higher homogeneity.	$\sum_{a,b}\frac{C(a,b)}{1+|a+b{|}^{2}}$
Sum average	Mean grey level sum distribution value.	∑ _*c*_*c C*_*a*+*b*_(*c*)
Sum entropy	Disorder of the sum distribution. Higher values of sum entropy indicate higher heterogeneity.	$-\sum_{c}C_{a+b}(c)\log C_{a+b}(c)$
Sum variance	Dispersion of values around the sum average. Higher values of sum variance relate to higher heterogeneity.	∑ _*c*_(*c* − *SA*)^2^ ∑ _*b*_*C*_*a*+*b*_(*c*)

#### 2.3.3 Local binary patterns descriptors

Local binary patterns (LBP) are another visual descriptor that quantify local signal variations. The original approximation characterises the way voxels relate to their neighbourhood using binary codes and summarises them for the entire region of interest using a histogram. The process is three-fold. First, the binary code for each voxel is computed by analysing the way its intensity relates to the ones of its neighbours: if the value is higher than the one of its neighbour, we assign a zero; and a one otherwise. For instance, if the voxel value is equal to 4 and the neighbouring intensities are 1 − 2 − 3 − 4 − 5 − 6 − 7 − 8, then the corresponding code would be 1 − 1 − 1 − 0 − 0 − 0 − 0 − 0. Second, the frequency of each one of these codes is totalised. Third, the resulting histogram is divided by its sum to express probability. The normalised histogram is used as a texture descriptor.

In this work, we considered two variants of the original approximation called uniform local binary patterns (ULBP) [28] and local configuration patterns (LCP) [29]. The former maps the original set of codes (2^8^ = 256 different binary patterns) to a subset of 59 codes to reduce the cardinality of the histogram and provide the descriptor with a simple rotation invariance. The latter combines the LBP with rotation invariance and another descriptor which quantifies local linear dependencies between a voxel and its neighbours. We calculated these descriptors for each region of interest for each patient using a radius equal to 1. For each time point and each region of interest, the number of ULBP and LCP features per patient were 59 and 81, in that order.

### 2.4 Functional principal component analysis

Comparing the resulting dynamic descriptors could be an intricate task due to the dimensionality of the problem:

• RPS: 128 features × 21 time points × 42 patients;
• GLCM: 9 features × 21 time points × 42 patients;
• ULBP: 59 features × 21 time points × 42 patients;
• LCP: 81 features × 21 time points × 42 patients.

Conventional feature reduction approaches, such as traditional principal component analysis (PCA) or auto-encoders, are not suitable for this problem since the data temporality would be neglected in principle. Therefore, we resorted to applying the functional principal component analysis (FPCA) method proposed by Happ and Greven [30] which allows us to model each descriptor as a function in time, reduce the time dimension respecting its nature, and obtain a single score per principal mode of variation. In a nutshell, the idea is to reduce the cardinality in one dimension (time) and then on another one (space). Each one of the elements of each descriptor can be seen as a function in time. In such a way, we could find the eigenvalues and eigenfunctions that better describe them. Let *D* be the number of elements under study, *P* = 42 the number of patients, and *R* = {*R*^(1)^,…,*R*^(*D*)^} the set of elements, each of them described by the corresponding measurements, $r_{1}^{\left(j\right)}$, $r_{2}^{\left(j\right)},\ldots ,r_{P}^{\left(j\right)}$, *j* = 1,…,*D*, the overall process is four-fold. First, each component was centred by subtracting its mean value. Second, eigenfunctions and scores were calculated for each component using the FPCA. The principal component functions were obtained constructively by finding orthogonal functions $\Phi_{k}^{\left(j\right)}$, *k* = 1,…,*M*^(*j*)^, for which principal component scores $\xi_{ik}^{\left(j\right)}$, *i* = 1,…,*P*, mathematically expressed as

(4) $$\xi_{ik}^{\left(j\right)}=\int \Phi_{k}^{\left(j\right)}(t)\;r_{i}^{\left(j\right)}(t)dt,$$

maximised $\sum_{i}{\xi_{ik}^{\left(j\right)}}^{2}$, subject to $||\Phi_{k}^{\left(j\right)}|{|}^{2}=1$. We set *M*^(*j*)^ to five as resulting eigenvectors accounting for the 99% of the univariate variation. Third, all of these scores $\xi_{ik}^{\left(j\right)}$ were arranged in a matrix form, $\Xi \in R^{P\times \sum M^{\left(j\right)}}$, such that the *i*th row contained $\left(\xi_{i1}^{\left(1\right)},\ldots ,\xi_{iM^{\left(1\right)}}^{\left(1\right)},\ldots ,\xi_{i1}^{\left(D\right)},\ldots ,\xi_{iM^{\left(D\right)}}^{\left(D\right)}\right)$. Fourth, scores were calculated using eigenanalysis on the covariance matrix of Ξ. We resorted to analysing the first mode of variation. The output was a single score per subject and per region of interest.

### 2.5 Validation against clinical parameters

We applied statistical tests to determine whether patients with similar health status exhibit similar principal component (PC) scores. We used the Kruskal-Wallis test for testing differences in PC scores between patients grouped by overall burden of SVD features. We considered multiple linear regression to establish whether age, WMH volume, biological sex, and stroke lesion type were associated with the PC scores (i.e. PC score = *β*_0_ + *β*_*Age*_ ⋅Age + *β*_*WMH*_ ⋅WMH vol + *β*_*Sex*_ ⋅Sex + *β*_*Lac*_ ⋅Lac, where each *β* ∈ℝ is a standardised regression coefficient).

### 2.6 Comparison against other techniques

We compared our proposal against two surrogate measures of small vessel dysfunction: area under the enhancement curve (AUEC) [31] and signal enhancement slope [32,33]. The idea behind both approaches is as follows. The former computes integral of the enhancement curve over time since higher accumulation of contrast agent leads to higher enhancement (thus, higher AUEC). The latter calculates the slope of the enhancement curve (assuming linearity after the bolus arrival peak) relates to the degree of contrast agent leakage. In both cases, we computed the enhancement curve on the sampling points described in Section 2.2, measured AUEC and slope, and used the statistical tests in Section 2.5 to establish the relationship between these measurements and clinical variables.

## 3 Experiments and results

We applied our framework to the 42 cases by segmenting each of the 42 DCE-MRI scans, sampling the signal in each region of interest, describing signal in each time point and region of interest, and applying FPCA on the resulting dynamic descriptions to analyse directions of variations within the data. We explored whether these variations were associated with clinical variables.

We grouped the PC scores by the total SVD score and applied the Kruskal-Wallis test to determine whether there were significant differences between groups. The results are displayed in Table 4. We observed significant differences using three descriptors: RPS extracted from blood vessels [low slice], deep GM, and WM [middle-high slice] (9.28 ≤ *χ*^2^ ≤ 15.56, *p*-value ≤ 0.05, *df* = 4); ULBP from WM [low-middle, middle-high, and high slices] (10.83 ≤ *χ*^2^ ≤ 11.45, *p*-value ≤ 0.05, *df* = 4), and LCP from blood vessels [low slice] and WM [middle-high slice] (*χ*^2^ = 9.69, *p*-value < 0.05, *df* = 4 and *χ*^2^ = 10.26, *p*-value < 0.05, *df* = 4, respectively). The other four descriptors did not vary significantly with the overall burden of SVD in any of the tissues in any of the four slices: signal enhancement, GLCM metrics, area under the enhancement curve, and enhancement curve slope.

**Table 4:** Kruskal-Wallis values obtained by grouping PC scores by total SVD score. The results are expressed concerning *χ*^2^ and Pr (*p*-value). The degrees of freedom were four. Enh, RPS, GLCM, ULBP, LCP, AUEC and slope stand for signal enhancement, radial power spectrum, grey co-occurrence matrix metrics, uniform local binary patterns, linear configuration model, area under enhancement curve, and enhancement curve slope, respectively. Values in bold are significant (p-value < 0.05).

Method		Low	Low-middle					Middle-high				High		
		BL	CSF	BL	GMD	GMC	WM	BL	CSF	GMC	WM	BL	GMC	WM
Enh	*χ*^2^	4.93	3.38	4.00	3.53	3.20	6.50	2.88	4.02	3.00	8.09	4.97	7.57	5.35
	Pr	0.29	0.50	0.41	0.47	0.52	0.16	0.58	0.40	0.56	0.09	0.29	0.11	0.25
RPS	*χ*^2^	**10.49**	7.69	2.32	**15.56**	8.35	5.87	5.91	7.54	**10.15**	**9.28**	6.58	6.64	8.97
	Pr	**0.03**	0.09	0.68	**0.01**	0.08	0.21	0.21	0.11	**0.04**	**0.05**	0.16	0.16	0.06
GLCM	*χ*^2^	2.32	4.70	4.55	2.44	5.03	6.01	3.46	5.52	2.65	6.50	0.44	0.92	6.06
	Pr	0.67	0.32	0.33	0.65	0.28	0.19	0.48	0.24	0.61	0.17	0.97	0.92	0.19
ULBP	*χ*^2^	4.31	5.16	1.00	4.41	3.25	**10.83**	2.17	2.52	2.72	**11.45**	1.06	6.54	**11.09**
	Pr	0.37	0.27	0.91	0.35	0.52	**0.03**	0.70	0.64	0.61	**0.02**	0.90	0.16	**0.03**
LCP	*χ*^2^	**9.69**	6.57	0.55	4.19	3.85	7.56	2.59	4.62	0.98	**10.26**	2.64	3.78	7.76
	Pr	**0.04**	0.16	0.97	0.38	0.43	0.11	0.63	0.33	0.91	**0.03**	0.62	0.44	0.10
AUEC	*χ*^2^	4.99	3.45	6.32	3.64	4.28	6.50	2.82	3.39	2.97	8.62	5.50	7.65	5.45
	Pr	0.29	0.49	0.18	0.46	0.37	0.16	0.59	0.49	0.56	0.07	0.24	0.11	0.24
Slope	*χ*^2^	4.40	3.30	4.60	1.62	2.35	1.61	6.39	5.44	7.98	2.29	4.08	2.26	1.66
	Pr	0.35	0.51	0.33	0.80	0.67	0.81	0.17	0.24	0.09	0.68	0.40	0.69	0.80

We carried out multiple linear regression to investigate whether biological sex, age, WMH volume, and stroke lesion type were associated with the observed PC score. The results are condensed in Table 5. Overall, we observed that (i) these four covariates influenced the features in blood vessels, CSF, deep GM, and WM, but not the ones on the cortical GM region, and (ii) the analysis of the signal enhancement slope did not result in significant associations.

**Table 5:** Multiple linear regression analysis between PC scores for each descriptor and for each region of interest as response variable and age, WMH volume, biological sex, and stroke lesion type as predictor variables. The regression results are expressed concerning adjusted *R*^2^, Pr (*p*-value), and *β* values. Enh, RPS, GLCM, ULBP, LCP, AUEC, and slope stand for signal enhancement, radial power spectrum, grey co-occurrence matrix metrics, uniform local binary patterns, linear configuration model, area under enhancement curve, and enhancement curve slope, respectively. Values in bold are significant (p-value < 0.05).

Method		Low	Low-middle					Middle-high				High		
		BL	CSF	BL	GMD	GMC	WM	BL	CSF	GMC	WM	BL	GMC	WM
Enh	*R*^2^	0.08	**0.19**	**0.14**	0.10	0.10	0.11	**0.20**	**0.19**	0.10	0.10	**0.18**	0.13	0.11
	Pr	0.14	**0.02**	**0.05**	0.10	0.10	0.09	**0.02**	**0.02**	0.11	0.09	**0.02**	0.06	0.08
	Pr_Age_	0.08	0.57	0.42	0.85	0.77	0.93	0.18	0.70	0.99	0.94	0.53	0.98	0.89
	Pr_WMH_	0.20	**0.02**	**0.05**	0.02	0.02	0.02	0.06	**0.00**	0.02	0.02	**0.03**	0.01	0.02
	Pr_Sex_	0.59	0.14	0.19	0.56	0.52	0.48	**0.04**	1.00	0.57	0.52	**0.05**	0.49	0.56
	Pr_Lac_	0.25	**0.03**	0.06	0.16	0.14	0.12	0.07	**0.05**	0.11	0.13	0.07	0.10	0.11
	*β*_Age_	−0.31	0.09	−0.14	−0.03	0.05	0.01	−0.22	0.06	0.00	0.01	−0.10	0.00	0.02
	*β*_WMH_	0.21	**0.38**	**0.31**	0.40	0.38	0.39	0.29	**0.46**	0.38	0.39	**0.35**	0.41	0.40
	*β*_Sex_	−0.08	−0.21	−0.20	−0.09	−0.10	−0.11	**−0.30**	0.00	−0.09	−0.10	**−0.29**	−0.10	−0.09
	*β*_Lac_	0.19	**0.35**	0.31	0.23	0.25	0.26	0.29	**0.32**	0.27	0.26	0.29	0.27	0.27

RPS	*R*^2^	**0.15**	**0.17**	0.07	**0.20**	0.11	**0.15**	0.11	0.04	0.07	**0.15**	**0.21**	0.12	**0.15**
	Pr	**0.04**	**0.03**	0.15	**0.01**	0.09	**0.04**	0.09	0.24	0.16	**0.04**	**0.01**	0.07	**0.05**
	Pr_Age_	0.22	0.24	0.12	0.59	0.07	0.50	0.15	0.30	0.86	0.60	0.03	0.78	0.65
	Pr_WMH_	**0.01**	**0.05**	0.09	**0.00**	0.06	**0.02**	0.09	0.16	0.06	**0.02**	**0.01**	0.04	**0.02**
	Pr_Sex_	0.25	0.14	0.27	0.09	0.38	0.09	0.66	0.58	0.36	0.10	0.73	0.16	0.11
	Pr_Lac_	0.17	**0.04**	0.40	0.13	0.38	0.15	0.11	0.16	0.11	0.16	0.12	0.06	0.15
	*β*_Age_	0.21	−0.19	0.28	−0.09	0.32	0.11	0.25	−0.18	−0.03	0.09	0.36	−0.05	0.08
	*β*_WMH_	**−0.40**	**−0.32**	−0.28	**0.45**	−0.30	**−0.37**	−0.28	0.23	−0.32	**−0.39**	**−0.39**	−0.33	**−0.39**
	*β*_Sex_	−0.17	0.22	0.17	0.25	−0.13	−0.26	0.07	−0.09	−0.14	−0.25	0.05	−0.21	−0.24
	*β*_Lac_	−0.22	**−0.33**	−0.14	0.24	−0.15	−0.24	−0.27	0.24	−0.28	−0.23	−0.24	−0.32	−0.24

GLCM	*R*^2^	−0.05	**0.24**	−0.06	0.01	−0.05	−0.05	0.00	−0.09	−0.02	**0.14**	−0.02	0.05	0.08
	Pr	0.72	**0.01**	0.79	0.39	0.75	0.69	0.40	0.94	0.52	**0.05**	0.55	0.22	0.13
	Pr_Age_	0.66	0.75	0.89	0.93	0.56	0.72	0.13	0.55	0.34	**0.01**	0.51	0.07	0.08
	Pr_WMH_	0.75	**0.02**	0.90	0.25	0.74	0.93	0.32	0.92	0.69	0.86	0.52	0.26	0.25
	Pr_Sex_	0.26	**0.01**	0.33	0.13	0.87	0.40	0.55	0.96	0.87	0.06	0.17	0.39	0.04
	Pr_Lac_	0.59	0.50	0.52	0.73	0.35	0.21	0.71	0.79	0.30	0.24	0.62	0.72	0.56
	*β*_Age_	0.08	−0.05	−0.02	−0.02	−0.11	−0.07	−0.28	−0.11	0.17	**−0.45**	−0.12	−0.33	−0.31
	*β*_WMH_	−0.06	**0.37**	−0.02	0.20	0.06	0.01	0.17	−0.02	−0.07	0.03	0.11	0.19	0.19
	*β*_Sex_	0.19	**0.40**	−0.16	0.24	0.03	−0.14	0.10	0.01	−0.03	−0.28	−0.23	0.13	−0.32
	*β*_Lac_	−0.10	−0.10	0.12	−0.06	0.17	−0.22	0.07	0.05	−0.18	−0.19	0.09	0.06	−0.10

ULBP	*R*^2^	0.08	**0.17**	−0.09	0.09	0.08	−0.06	−0.02	−0.06	0.01	0.13	−0.04	−0.08	**0.25**
	Pr	0.14	**0.03**	0.96	0.12	0.14	0.80	0.55	0.79	0.37	0.06	0.66	0.91	**0.01**
	Pr_Age_	0.54	0.79	0.93	0.23	0.09	0.79	0.14	0.77	0.18	0.40	0.99	0.72	0.26
	Pr_WMH_	0.20	**0.04**	0.90	0.54	0.76	0.47	0.43	0.40	0.52	0.01	0.48	0.48	**0.01**
	Pr_Sex_	0.04	**0.05**	0.50	0.06	0.39	0.52	0.69	0.33	0.39	0.28	0.36	0.70	**0.02**
	Pr_Lac_	0.45	**0.05**	0.73	0.67	0.41	0.38	0.24	0.87	0.13	0.55	0.45	0.64	0.54
	*β*_Age_	−0.11	−0.04	−0.02	−0.21	−0.30	−0.05	0.27	−0.05	−0.25	0.14	−0.00	0.07	−0.18
	*β*_WMH_	−0.21	**−0.32**	0.02	−0.10	−0.05	−0.12	−0.13	0.15	−0.11	−0.45	−0.12	−0.12	**−0.40**
	*β*_Sex_	0.32	**0.29**	0.11	0.29	−0.13	−0.11	0.06	−0.16	−0.14	−0.16	−0.15	0.06	**−0.33**
	*β*_Lac_	−0.13	**−0.32**	−0.06	0.07	0.14	−0.16	0.21	0.03	−0.27	−0.10	0.14	−0.09	−0.09

LCP	*R*^2^	−0.01	**0.20**	−0.07	0.11	0.05	−0.06	−0.02	−0.03	−0.06	**0.25**	−0.02	−0.08	**0.36**
	Pr	0.47	**0.02**	0.86	0.09	0.22	0.78	0.53	0.59	0.80	**0.01**	0.54	0.92	**0.00**
	Pr_Age_	0.19	0.36	0.46	0.02	0.15	0.92	0.19	0.67	1.00	0.47	0.93	0.41	0.15
	Pr_WMH_	0.45	**0.03**	0.75	0.39	0.79	0.25	0.26	0.36	0.93	**0.00**	0.78	0.57	**0.00**
	Pr_Sex_	0.24	**0.05**	0.48	0.15	0.18	0.82	0.98	0.17	0.96	0.15	0.39	0.78	**0.01**
	Pr_Lac_	0.28	0.06	0.80	0.79	0.41	0.89	0.27	0.81	0.26	0.69	0.21	0.85	0.71
	*β*_Age_	−0.24	−0.15	0.14	−0.4	−0.25	0.02	0.25	0.08	0.00	0.11	0.02	−0.15	0.21
	*β*_WMH_	0.13	**−0.34**	−0.05	0.14	0.04	0.20	−0.19	−0.16	0.02	**−0.56**	0.05	0.10	**0.47**
	*β*_Sex_	−0.19	**0.29**	0.12	0.22	−0.21	0.04	0.00	0.22	−0.01	−0.20	0.14	0.05	**0.35**
	*β*_Lac_	−0.19	−0.30	−0.05	−0.04	0.14	−0.02	0.20	−0.04	−0.21	−0.06	−0.22	−0.03	0.05

AUEC	*R*^2^	0.10	**0.20**	0.12	0.10	0.11	0.11	**0.21**	**0.19**	0.10	0.10	**0.19**	0.13	0.11
	*Pr*	0.10	**0.02**	0.08	0.10	0.08	0.09	**0.01**	**0.02**	0.10	0.09	**0.02**	0.06	0.08
	Pr_Age_	0.06	0.57	0.48	0.85	0.87	0.94	0.17	0.70	1.00	0.94	0.52	0.99	0.89
	Pr_WMH_	0.14	**0.02**	0.07	0.02	0.01	0.02	0.06	**0.00**	0.02	0.02	**0.02**	0.01	0.01
	Pr_Sex_	0.58	0.14	0.05	0.56	0.61	0.48	**0.04**	0.99	0.57	0.52	**0.05**	0.49	0.55
	Pr_Lac_	0.25	**0.03**	0.26	0.16	0.13	0.12	0.07	**0.04**	0.10	0.13	0.06	0.10	0.11
	*β*_Age_	0.33	−0.09	0.12	0.03	0.03	−0.01	0.22	−0.06	0.00	−0.01	0.11	−0.00	−0.02
	*β*_WMH_	−0.24	**−0.38**	−0.30	−0.40	−0.41	−0.39	−0.29	**−0.46**	−0.38	−0.39	**−0.36**	−0.41	−0.40
	*β*_Sex_	0.08	0.21	0.31	0.09	0.08	0.11	**0.31**	−0.00	0.09	0.10	**0.29**	0.10	0.09
	*β*_Lac_	−0.19	**−0.36**	−0.18	−0.24	−0.25	−0.26	−0.29	**−0.33**	−0.27	−0.26	−0.30	−0.27	−0.27

Slope	*R*^2^	0.02	0.01	−0.09	−0.05	−0.02	0.04	0.05	−0.00	0.02	0.10	0.03	0.07	0.01
	*Pr*	0.33	0.35	0.93	0.69	0.51	0.26	0.20	0.42	0.34	0.10	0.28	0.16	0.39
	Pr_Age_	0.14	0.54	0.93	0.29	0.18	0.67	0.48	0.91	0.24	0.07	0.69	0.01	0.34
	Pr_WMH_	0.93	0.44	0.88	0.33	0.50	0.68	0.37	0.21	0.32	0.39	0.24	0.59	0.68
	Pr_Sex_	0.60	0.13	0.86	0.53	0.30	0.92	0.34	0.67	0.10	0.05	0.47	0.73	0.13
	Pr_Lac_	0.55	0.71	0.40	0.97	0.62	0.04	0.09	0.26	0.98	0.39	0.07	0.22	0.35
	*β*_Age_	−0.27	−0.11	0.02	−0.20	−0.25	−0.08	−0.12	0.02	−0.21	−0.32	0.07	−0.46	−0.17
	*β*_WMH_	−0.01	−0.13	0.03	0.17	0.11	0.07	0.15	−0.22	0.17	0.14	0.20	0.09	0.07
	*β*_Sex_	0.08	0.24	0.03	0.10	0.17	−0.02	−0.15	0.07	−0.27	0.30	−0.11	−0.05	0.25
	*β*_Lac_	0.10	0.07	0.16	−0.01	−0.09	−0.37	0.30	0.20	0.00	−0.14	0.32	−0.21	−0.16

Concerning CSF, the regression results indicate that the resulting models explained between a fifth and a fourth of the variance and were significant predictors of the observed PC scores (*p*-value ≤ 0.05) in six of evaluated descriptors: signal enhancement (both low-middle and middle-high slices), RPS (only low-middle slice), GLCM metrics (only low-middle slice), ULBP (only low-middle slice), LCP (only low-middle slice), and AUEC (both low-middle and middle-high slices). Three out of four covariates, WMH volume, sex, and stroke lesion type, were relevant predictors of the observed intensity, spectral, texture features measured in the CSF region. WMH volume was a strong predictor in all six models: signal enhancement (*β* = 0.38;*p*-value < 0.05), RPS (*β* = −0.32;p-value = 0.05), GLCM metrics (*β* = 0.37;*p*-value < 0.05), ULBP (*β* = −0.32;*p*-value < 0.05), LCP (*β* = −0.34;*p*-value < 0.05), and AUEC (*β* = −0.38;*p*-value < 0.05). While biological sex contributed significantly to models built on GLCM metrics (*β* = 0.40;*p*-value < 0.05), ULBP (*β* = 0.29;*p*-value = 0.05), LCP (*β* = 0.29;*p*-value = 0.05), stroke lesion type appeared to be significant predictors in the models built on RPS (*β* = −0.33;*p*-value < 0.05), ULBP (*β* = −0.32;*p*-value = 0.05), AUEC (*β* = −0.36;*p*-value < 0.05). The age of the patients did not seem to influence considerably the features observed in any of the models.

Regarding blood vessels (carotid arteries, basilar artery, sagittal sinus), the regression results implied strong relationships health status and observed intensity and spectral features (signal enhancement, RPS, and AUEC), explaining approximately 20% of the data variation and being significant predictors of these models (*p*-value < 0.05). Signal enhancement values were associated to the overall burden of WMH in low-middle (*β* = 0.31;*p*-value = 0.05) and high (*β* = 0.35;*p*-value < 0.05) slices and the biological sex of the patients in middle-high (*β* = −0.30;*p*-value < 0.05) and high (*β* = −0.29;*p*-value = 0.05) slices. These associations were similar for AUEC, except that the features measured in the low-middle slices were not significant (*p*-value > 0.05).

In the low-middle slice, the overall burden of WMH contributed significantly to the observed spectral features in deep GM (*β* = 0.45;*p*-value < 0.01) and WM (*β* = −0.37;*p*-value < 0.05), describing approximately 20% and 15% of their variability. However, no other descriptor seemed to encode any relevant information for these two tissues in this slice.

Only spectral and textural features measured in the WM region in the middle-high and/or high slices displayed relevant relationships with health status. Model covariates explained between 14% and 36% of the variance of RPS, GLCM metrics, ULBP, and LCP features. WMH volume contributed significantly to the RPS features in both slices (*β* = −0.39;*p*-value < 0.05 and *β* = −0.39;*p*-value < 0.05, respectively), ULBP in the high slice (*β* = −0.40;*p*-value < 0.05), LCP in the both slices (*β* = −0.56;*p*-value < 0.01 and *β* = 0.47;*p*-value < 0.01, respectively). Biological sex predicted significantly the features of two descriptors, ULBP (*β* = −0.33;*p*-value < 0.05) and LCP (*β* = 0.35;*p*-value < 0.05). Age significantly influenced the values of GLCM (*β* = −0.45;*p*-value < 0.05). Stroke lesion type did not appear relevant in any of the models built with data from middle-high or high slices.

## 4 Discussion

In this paper, we propose a framework incorporating dynamic spectral and textural descriptors and functional principal component analysis to study dynamic brain MRI signals of brain pathology. In particular, we applied our processing pipeline to the study of SVD tissue changes using DCE-MRI acquisitions. We analysed the dynamic descriptors from blood vessels, CSF, grey and white matter brain regions of a population with features of SVD of differing extents to examine whether subjects with different biological sex, age, WMH volume, stroke lesion type, and overall load of SVD features exhibited distinctive patterns. To the best of our knowledge, this is the first time that these dynamic descriptors have been examined jointly for this purpose. Of note, our framework could be used for analysing other dynamic and non-dynamic brain MR acquisitions, with further testing.

Most of the features extracted from the CSF cavities near the choroid plexus (low-middle slice) were significantly associated with the biological sex or stroke lesion type, and the overall burden of WMH. We observed that the overall enhancement in the CSF cavities increased if patients were female or had a lacunar stroke, and with decreasing WMH burden. In accordance with previous findings [10], we observed a relationship between burden of features of SVD and leakage of Gadolinium-based contrast agent into CSF. However, our results suggest that the CSF enhancement might be inversely proportional to the burden of WMH. Bigger sample size and further testing are needed to determine the direction of the relationship. Our framework found features correlated with WMH burden and, as increased WMH load is known to be associated with blood-brain barrier leakage [9], our proposal shows promise for studying subtle small vessel dysfunction.

We observed that surrogate measures of small vessel disruption extracted from the signal enhancement curve, such as area under the enhancement curve, could capture relevant information linked with the health status of the patients, but some of the texture and spectral descriptors were more sensitive to variations in the deep GM or WM. In fact, we noticed that while both the slope and area under the enhancement curve measurements in WM did not reflect the health status significantly, but spectral and texture descriptors did. This might be a consequence of spectral and texture descriptors taking into account and encoding neighbouring relationships. The power spectrum reflected information associated with overall WMH burden in blood vessels, CSF, deep GM, and WM; being an encouraging outcome since it shows the analysis of the power spectra is more descriptive than current semi-quantitative surrogates of dysfunction.

The current proposal exhibits two drawbacks: the region sampling strategy and the generalisability. First, region sampling prevents descriptors from encoding volumetric information, but sample selection is tedious and not resilient to motion. Due to the prolonged scanning process, brain DCE-MRI acquisitions are prone to head motion artefacts. In small vessel dysfunction assessments, imaging artefacts confound whether tenuous enhancements are a consequence of subtle blood-brain barrier abnormalities and, although the repercussions of motion have not been documented for this particular problem, we believe that they compromise both the interpretation and subsequent result interpretation. Notwithstanding that we realigned all time points to the pre-contrast scans to correct for bulk patient movement, this approximation does not compensate for possible information loss or k-space dephasing due to motion. Second, the imaging protocol influences the features that are captured by the different descriptors as none of them is scale-invariant in principle and also the synthesis step as lower temporal resolution results in less information in the time domain. Even though the acquisition protocol was fixed in this study, their application to multi-centre studies might be restricted.

Future work should contemplate finding automatically anatomically-relevant regions proximal to arterial territories or in which signal to noise ratios are the highest. In such a way, we could enlarge our sample size to (i) draw stronger conclusions, (ii) establish clearer links between computer vision descriptors and underlying physiopathological processes, and (iii) determine whether these descriptors are useful in dysfunction classification problems. Additionally, the current proposal needs to be tested on diagnostic sequences and pre- vs post-contrast analyses to examine whether they capture dysfunction-related information.

Our findings add confidence to previous studies in which DCE-MRI signals from patients with different age, health status, and premorbid brain condition exhibited different tendencies [26,33]. Our proposed framework seems promising and feasible, but it needs further testing on a larger sample and on pre- vs post-contrast and cross-sectional studies.

## Declaration of competing interest

None.

## Footnotes

1 Approximately 16 million according to figures presented in [4], but possibly 22.5 million following the latest World Health Organisation report on dementia [5].
